# Plasma Rich in Growth Factors in Bone Regeneration: The Proximity to the Clot as a Differential Factor in Osteoblast Cell Behaviour

**DOI:** 10.3390/dj12050122

**Published:** 2024-04-24

**Authors:** Eduardo Anitua, Mar Zalduendo, Roberto Tierno, Mohammad Hamdan Alkhraisat

**Affiliations:** 1 BTI-Biotechnology Institute, 01007 Vitoria, Spain; marimar.zalduendo@bti-implant.es (M.Z.); roberto.tierno@bti-implant.es (R.T.); mohammad.hamdan@bti-implant.es (M.H.A.); 2 University Institute for Regenerative Medicine & Oral Implantology, UIRMI (UPV/EHU-Fundación Eduardo Anitua), 01007 Vitoria, Spain

**Keywords:** PRGF, osteogenesis, *Runx2*, *SP7*, alkaline phosphatase

## Abstract

The osteogenic differentiation process, by which bone marrow mesenchymal stem cells and osteoprogenitors transform into osteoblasts, is regulated by several growth factors, cytokines, and hormones. Plasma Rich in Growth Factors (PRGF) is a blood-derived preparation consisting of a plethora of bioactive molecules, also susceptible to containing epigenetic factors such as ncRNAs and EVs, that stimulates tissue regeneration. The aim of this study was to investigate the effect of the PRGF clot formulation on osteogenic differentiation. Firstly, osteoblast cells were isolated and characterised. The proliferation of bone cells cultured onto PRGF clots or treated with PRGF supernatant was determined. Moreover, the gene expression of *Runx2* (ID: 860), *SP7* (ID: 121340), and *ALPL* (ID: 249) was analysed by one-step real-time quantitative polymerase chain reaction (RT-qPCR). Additionally, alkaline phosphatase (ALPL) activity determination was performed. The highest proliferative effect was achieved by the PRGF supernatant in all the study periods analysed. Concerning gene expression, the logRGE of *Runx2* increased significantly in osteoblasts cultured with PRGF formulations compared with the control group, while that of *SP7* increased significantly in osteoblasts grown on the PRGF clots. On the other hand, despite the fact that the PRGF supernatant induced *ALPL* up-regulation, significantly higher enzyme activity was detected for the PRGF clots in comparison with the supernatant formulation. According to our results, contact with the PRGF clot could promote a more advanced phase in the osteogenic process, associated to higher levels of ALPL activity. Furthermore, the PRGF clot releasate stimulated a higher proliferation rate in addition to reduced *SP7* expression in the cells located at a distant ubication, leading to a less mature osteoblast stage. Thus, the spatial relationship between the PRGF clot and the osteoprogenitors cells could be a factor that influences regenerative outcomes.

## 1. Introduction

Osteogenesis, or bone ossification, is the process that involves the formation of bone tissue by osteoblasts. Two osteogenic pathways have been described, endochondral or intramembranous ossification, both sharing mesenchymal stem cells (MSCs) as precursors [1]. Osteogenesis is controlled by a wide range of regulators and extracellular signalling pathways [2,3,4]. Firstly, the commitment to the bone lineage is guided by the activation of runt-related gene 2 (*Runx2*) transcription factor. Subsequently, preosteoblasts undergo a three-stage differentiation process with differential expression of well-stablished bone cell markers. Thus, the expression of the master transcription factor *Runx2*, up-regulated in the preosteoblast population, is steadily reduced during the osteoblast maturation process. Following *Runx2* activation, *SP7* (also named Zinc finger protein osterix) is expressed to support the progression of osteogenesis. Alkaline phosphatase (*ALPL*), collagen α1 (*Collα1*), bone morphogenetic proteins (BMPs), and osteopontin (*OPN*) are expressed in the early stages of osteoblast differentiation, while osteocalcin (*OC)* is associated with extracellular matrix (ECM) mineralization conducted by mature osteoblasts [5,6]. 

Numerous growth factors, cytokines and hormones have been classified as regulators of the osteogenic process that modulate several signalling pathways, like the Mitogen-Activated Protein Kinase (MAPK), Protein Kinase B (PKB/AKT), Wnt-ß-Catenin, and Notch pathways [4,7]. Certain growth factors, including BMP-2, BMP-4, BMP-6, BMP-7, and BMP-9, promote osteoblastic differentiation and bone formation [8,9,10]. In addition, three members of the fibroblast growth factor (FGF) family, FGF-2, FGF-9, and FGF-18, are also involved in the control of endochondral and intramembranous ossification [8]. Insuline-like Growth Factor (IGF-1), a protein found in the bone matrix, has been suggested to be involved in bone remodelling [11,12]. The role of VEGF and PDGF expression in stimulating bone marrow mesenchymal stem cell (BMSC) differentiation has also been described [13]. Moreover, several cytokines (like Interleukin IL-11 [14,15]), vitamin D [16,17], and hormones have been reported as modulators of the osteogenic process. The parathyroid hormone (PTH) is one of the key upstream regulators of Runx2 in such a way that bone resorption occurs if the PTH level is continuously elevated, and osteoblast differentiation is stimulated when the hormone levels increase in a discontinuous mode [18,19,20].

It is important to take into consideration that osteogenesis is affected by epigenetic modifications that alter the architecture of chromatin, such as DNA methylation or post-translational modification (PTM) in histone proteins (mainly methylation, phosphorylation, acetylation, and ubiquitylation) [21,22]. Recently, the involvement of non-coding RNAs (ncRNAs), including micro RNAs (miRNAs), long non-coding RNAs (lncRNAs), and circular RNAs (cirRNAs), has been highlighted [23]. miRNAs mediate bone ossification via the silencing or degrading of osteogenesis-related factors [24]. On the other hand, lncRNAs control messenger RNA (mRNA) transcription and translation, post-translational modifications, and the generation of small proteins and other smaller nc-RNAs [25,26]. The function of cirRNAs is still mostly obscure, but it seems to be related to enhanced osteoblast differentiation [27]. Finally, extracellular vesicles (EVs) are emerging as relevant vehicles for cell–cell communication. They shuttle a wide range of signalling molecules, including ncRNAs, mRNAs, and specific membrane or intra-vesicular proteins [28,29]. Bone matrix EVs are anchored to the ECM and contain phosphatases, annexins, and other potentially pro-osteogenic components, such as α5β1 integrin [30,31]. In this sense, they are suggested to be involved in the osteogenic differentiation of MSCs by up-regulating Runx2 and BMP-2 osteogenic markers [32].

Plasma Rich in Growth Factors (PRGF) is a blood-derived preparation where platelets are moderately concentrated in leukocyte-free plasma. PRGF formulations are naturally enriched in growth factors and are also susceptible to containing epigenetic factors such as ncRNAs and EVs [33,34,35,36]. PRGF has been tested in the cell culturing of periosteal cells, alveolar bone-derived osteoblasts, and human dental pulp stem cells [37,38,39,40]. The outcomes of these studies highlighted enhancements in cell proliferation, chemotaxis, migration, and osteogenic differentiation and mineralization. Several studies testing the involvement of other types of platelet-rich plasma (PRP) in the osteogenic process have been carried out [7,13,41,42,43,44]. In bone regeneration, PRGF is converted into a clot by the activation of platelets and coagulation cascade proteins [45,46,47]. The clot is then inserted into the bone defect to promote bone regeneration [48,49]. The growth factors and biomolecules released from the clots get diffused into the tissue and thus exert an effect on the cells that are not in direct contact with the clot [50,51,52]. However, there is a lack of studies simulating this biological scenario during bone regeneration. Therefore, the aim of this research was to evaluate the PRGF clot as a potential modulator of the osteogenic process in both osteoblast cells in close contact with it and in those at distant locations. In the latter case, the cell culture medium was supplemented with PRGF supernatant mimicking the releasate that would reach the remote cells.

## 2. Material and Methods

This study was performed by following the principles established in the Declaration of Helsinki of 1964 as revised in 2013 and also in accordance with the ethical standards of the Araba University Hospital Clinical Research Ethical Committee (this research was assessed and approved in September 2019; FIBEA-04-EP/17/Fresado bajas revoluciones). 

### 2.1. PRGF Preparation and Haematological Characterisation

After informed consent was signed, blood from one healthy donor was harvested into 3.8% (wt/v) trisodium citrate containing tubes. Blood was centrifuged for 8 min at 580× *g* (KMU15; BTI Biotechnology Institute, S.L., Vitoria, Spain). Subsequently, the whole plasma column just above the buffy coat was collected to obtain the PRGF preparation. 

Platelet, erythrocyte, and leukocyte concentrations were determined in the whole blood and PRGF samples by using a haematology analyser (Pentra ES 60; Horiba ABX, Montpellier, France).

**Figure 1:**
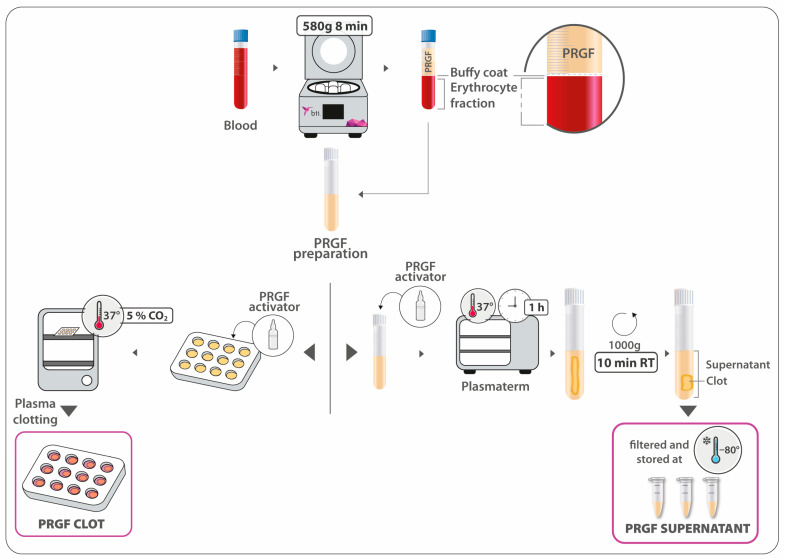
A schematic representation of the process of obtaining the two PRGF formulations: the PRGF clot and the PRGF supernatant.

With the aim of evaluating the effect of PRGF on in vitro osteogenic differentiation, two formulations of PRGF were used for cell treatment: PRGF clot and PRGF supernatant, as the releasate from the former formulation. PRGF preparation was activated into multi-well culture plates by mixing 10% calcium chloride (PRGF activator; BTI Biotechnology Institute, Vitoria, Spain) (20 µL CaCl_2_ 10% [m/v]: 1 mL PRGF ratio) and left to clot in a cell incubator at 37 °C in 5% CO_2_ atmosphere. Immediately after plasma clotting, the corresponding culture medium was added in order to avoid clot retraction. Finally, osteoblast cells were seeded onto the PRGF clot. For the obtention of the supernatant, the PRGF preparation was similarly activated. After leaving the PRGF clot for 1 h at 37 °C, it was centrifuged at 1000× *g* for 10 min at RT. The supernatant was then collected, filtered, aliquoted, and stored at −80 °C (Figure 1).

### 2.2. Osteoblast Cell Isolation

**Figure 2:**
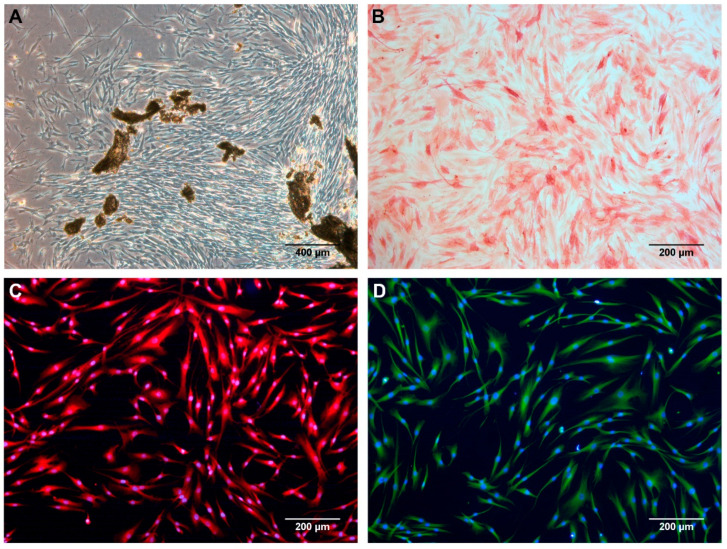
Isolation and characterisation of alveolar bone cells. (**A**) Bone cells were isolated by the explant method. (**B**) The alkaline phosphatase activity and the presence of (**C**) osteocalcin and (**D**) osteopontin osteoblast markers were used for cell characterisation.

Primary human alveolar osteoblasts were obtained from drilling bone particles that were generated during dental implant surgery of one patient, after the informed consent form was received and signed. Briefly, harvested alveolar bone was gathered in phosphate-buffered saline with 50 mg/mL gentamicin and 2.5 mg/mL amphotericin B (both from Sigma-Aldrich Inc., St. Louis, MO, USA) by using dental drills. The bone tissue was cultured as explants in osteoblast culture medium (ObM) enriched with growth supplements (ScienCell Research Laboratories Inc., Carlsbad, CA, USA), 5% foetal bovine serum (FBS) (*v*/*v*) (Biochrom AG, Leonorenstr, Berlin, Germany), 50 mg/mL gentamicin, and 2.5 mg/mL amphotericin B. Culture medium was changed 2 times a week. Cells leaving the explants were detached with animal origin-free trypsin-like enzyme (Gibco-Invitrogen, Grand Island, NY, USA) when they covered the culture surface entirely (Figure 2A). Trypan blue dye exclusion (Sigma-Aldrich) was used for cell viability assessment. From the first subculture onwards, primary osteoblasts were maintained in ObM supplemented with 15% FBS (*v*/*v*) and 50 mg/mL gentamicin (hereafter, ObGM, osteoblast growth culture medium). Cells between the 4th and 6th passages were used in the experiments.

### 2.3. Osteoblast Cell Characterisation

After cell culture amplification, bone cells were characterised by immunocytochemistry techniques [53,54,55]. ALPL activity was tested, as this enzyme is involved in bone matrix mineralization. Following the manufacturer’s instructions, bone cells were fixed with a citrate solution and then incubated in an alkaline dye mixture (Alkaline Phosphatase detection kit; Sigma-Aldrich).

The expression of 2 osteoblast markers was also analysed. Thus, isolated alveolar bone cells were fixed in a 3:1 mixture of methanol: acetic acid, and subjected to protein blocking for avoiding unspecific binding. Then, incubations with antibodies against osteopontin (Sigma-Aldrich) and osteocalcin (Origene, Rockville, MD, USA) and, subsequently, with the corresponding secondary antibodies (Molecular Probes, Eugene, OR, USA) were carried out. Finally, cell nuclei were highlighted by using Hoechst 33,342 (Molecular probes).

### 2.4. Osteoblast Proliferation

For testing the proliferative effect of both PRGF preparations, cells were seeded into 96-well culture plates at a density of 4000 cells/cm^2^, either on a bare plastic surface or onto 150 µL PRGF clots. Cells were suspended in ObGM in which FBS was replaced by PRGF supernatant in the first condition, while the culture medium used was unmodified ObGM when cells were seeded on PRGF clots. Osteoblasts were treated for 3, 7, and 10 days, and the culture medium was changed 2 times a week. Cells maintained with ObGM on plastic surface were used as positive controls. Three replicates of each condition were analysed. After each study period, cell proliferation was quantified by using the WST (tetrazolium salt, 4-[3-(4-iodophenyl)-2-(4-nitrophenyl)-2H-5-tetrazolio]-1,3-benzene disulfonate) colorimetric assay (Sigma-Aldrich). Following the manufacturer’s instructions, cells were incubated with WST reagent at 37 °C for 1 h, and absorbance was measured at 450/620 nm. Absorbance data were directly proportional to the number of living cells. Results for PRGF treatments were referred to those obtained for positive control and expressed in fold increase.

### 2.5. Osteoblast Gene Expression and Enzyme Activity Determination

#### 2.5.1. Cell Culture

Similarly to the cell proliferation assay, 4000 cells/cm^2^ were seeded either on a bare plastic surface or onto PRGF clots, depending on the treatment. Osteoblasts seeded directly on the plastic surface were treated with ObGM (control) or ObGM with 15% PRGF supernatant (*v*/*v*) instead of FBS. In addition, after forming PRGF clots into 12-well plates, cells were seeded onto them and cultured with ObGM. Thus, 1590 µL of PRGF was clotted, and immediately, 900 µL of the corresponding culture medium with alveolar bone cells was added on it. The same volume was added in the case of cells cultured on the plastic surface. Four replicates of the 2 treatments and control were assayed. Cell culture medium was changed on the 4th day. For ALPL activity determination, the culture media conditioned by osteoblast cells were collected on the 4th and 8th days from all the wells of the 3 different culture conditions. Furthermore, bone cells corresponding to the 4 replicates of each condition were also processed for gene expression analysis at the final time point.

#### 2.5.2. RNA Extraction and Quality Control (QC) Assessment

For the osteoblast gene expression analysis, total RNA was extracted from cultured bone cells by using the NucleoSpin RNA purification kit (Macherey-Nagel, Düren, Germany), following the manufacturer’s instructions. The concentration and purity of RNA extracts were determined by using a Biophotometer Plus spectrophotometer (Eppendorf, Hamburg, Germany).

#### 2.5.3. RT-qPCR Assays

A total of three genes related to bone metabolism and mineralization were included in the present study: *Runx2* (ID: 860), *SP7* (ID: 121340), and *ALPL* (ID: 249). One-step real-time quantitative polymerase chain reaction (RT-qPCR) was performed by using the Reliance Multiplex RT-qPCR Supermix combined with the following Prime PCR probe assays (BioRad, Hercules, CA, USA): *Runx2* (qHsaCEP0051329), *SP7* (qHsaCEP0025867), and *ALPL* (qHsaCEP0053252). As suggested by Abuna et al. [56], Eif2b1 (qHsaCIP0030434) and YWHAZ (qHsaCIP0029093) were selected as reference genes, considering a wide range of algorithms for assessing expression stability in osteoblasts. Assays were conducted in a CFX96 Touch Real-Time PCR Detection System (BioRad, Hercules, CA, USA), and the thermal cycling conditions were 50 °C (10 min) for reverse transcription, 95 °C (10 min) for DNA polymerase activation and template denaturation, and 40 cycles at 95 °C (10 s) and 60 °C (30 s) in a 10 µL reaction volume, according to manufacturer’s recommendations. For each biological sample, a total of three technical replicates were included. Multiplexing was validated experimentally after comparing the Cq values and amplification efficiencies of multiplex and singleplex reactions. For RT-qPCR efficiency calculation, five 5-fold serial dilution assays were performed, and the standard curve method was applied [57].

#### 2.5.4. Gene Expression Analyses

Relative gene expression normalized via the geometric averaging of multiple internal control genes was estimated according to the approaches described by Vandesompele et al. [58] and Hellemans et al. [59]. Control culture conditions (treatment with ObGM and culturing on plastic surface) were used as calibrator for calculating ∆Ct values. After checking assumptions for parametric statistical procedures, the rank-based Kruskal–Wallis non-parametric test was applied to log-transformed relative gene expression data [60]. When significant differences were detected between medians of different groups, post hoc testing was performed via Dunn’s pairwise test for multiple comparisons of mean rank sums with standard culture conditions as the control group [61] adjusted by using the Benjamini–Hochberg procedure [62]. Statistical analyses and data visualization were conducted by using the R v4.1.1 [63], including the PMCMRplus package [64], and Python v3.10.5 [65] computing environments. In a similar way to the proliferation assays, the results were expressed as relative units compared with the control. The reason behind this decision was to facilitate the interpretation of the results by equating the representation technique used to show all the analysed variables. This is a common practice for representing relative gene expression data, since in most cases, changes in gene expression in treatments are analysed in relation to another reference sample (calibrator or untreated control), regardless of the method used to quantify the relative gene expression [58,66,67]. In this sense, absolute gene expression values are not measured, but rather fold changes, 2^−ΔΔCt^, or gene expression levels in relation to the calibrator or untreated/control sample are, so this control sample is not usually represented because their average normalized relative quantity is always zero (or one if results are expressed as logarithm).

#### 2.5.5. Enzymatic Activity Determination

Culture media conditioned by osteoblast cells were collected 4 and 8 days after seeding the cells. In order to quantify the enzymatic activity per cell culture (well) under different conditions, the volume recovered from each well was measured. After centrifugation at 1000× *g* and RT for 10 min, it was aliquoted and stored at −80 °C until usage for the determinations.

For ALPL enzymatic activity quantification, an ultra-sensitive fluorometric assay was used (Abcam, Cambridge, UK). The fluorescent signal resulting from the cleavage of the phosphate group of the non-fluorescent substrate by ALPL was determined.

The results obtained for PRGF treatments were expressed as relative units compared with the control (normal growing conditions: culturing on bare plastic surface with ObM supplemented with 15% FBS).

## 3. Results

### 3.1. PRGF Preparation and Haematological Characterisation

PRGF preparation was characterised in terms of cellular composition (Table 1). The platelet content was found to be 2.2-fold higher than in whole blood, with almost no leukocytes or erythrocytes detected. 

### 3.2. Osteoblast Cell Characterisation

Alkaline phosphatase activity was detected in all the cells isolated from the bone particles (Figure 2B). Furthermore, the immunofluorescence detection of osteocalcin and osteopontin resulted in an entirely positive labelling of alveolar bone-derived cells regarding both biochemical osteoblastic markers (Figure 2C,D). Considering that no Hoechst-stained nuclei were detected without being associated with the labelling of any of the two proteins, it could be affirmed that almost all cells were positive for osteocalcin and osteopontin bone markers. Hence, the results from both characterisation assays confirmed the osteoblastic nature of the isolated cells. 

### 3.3. Osteoblast Proliferation

**Figure 3:**
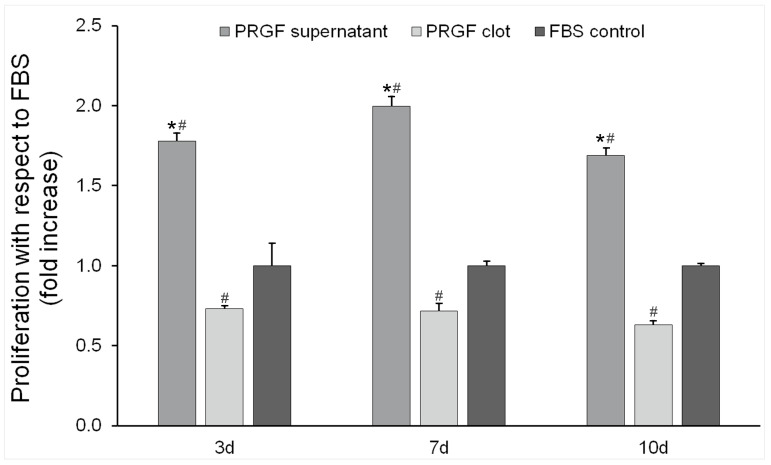
Cell proliferation assay. Osteoblast cells were treated with PRGF clot and PRGF supernatant formulations for 3, 7, and 10 days (n = 4). Results were expressed as fold increases with respect to the proliferation achieved in the control condition (osteoblasts cultured on bare plastic surface with ObM supplemented with 15% FBS (*v*/*v*)). * Statistically significant differences with respect to control condition for the same study period (*p* ≤ 0.05). ^#^ Statistically significant differences with respect to PRGF clot for the same study period (*p* ≤ 0.05).

The effect of both PRGF clot and supernatant formulations on osteoblast cell proliferation was tested on days 3, 7, and 10. Control cultures on a plastic surface with ObGM were included as optimal growing conditions. The results were expressed as relative units (percentage) compared with the control condition at each point in time analysed. The highest proliferative effect was achieved by the PRGF supernatant in all the study periods analysed, as it is shown in Figure 3 (1.78x ± 0.05 vs. 0.73x ± 0.02, 2.00x ± 0.06 vs. 0.72x ± 0.05, and 1.69x ± 0.05 vs. 0.63x ± 0.03, for supernatant vs. clot, respectively, on days 3, 7, and 10). The differences obtained for PRGF clot treatment with respect to the control were maintained during all the study period. In the case of the supernatant, the differences increased after 7 days. Also worth mentioning is the superconfluent state of the alveolar bone cell culture observed on day 10 in the PRGF supernatant treatment group, which is why the proliferation relative to the control induced by this formulation at this time decreased compared with that of the previous study time point.

### 3.4. Osteoblast Gene Expression and ALPL Activity Quantification

#### 3.4.1. Osteoblast Gene Expression

**Figure 4:**
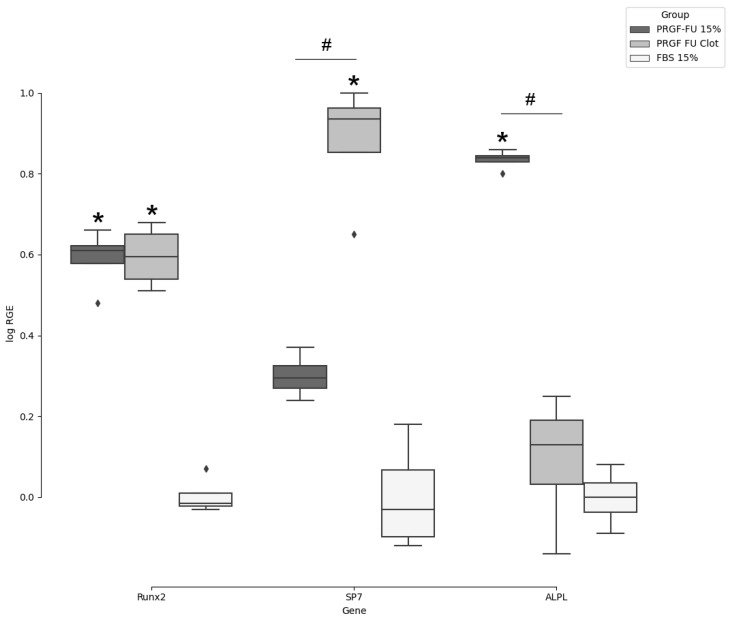
Box plot for gene expression levels (log RGE) of *Runx2*, *SP7*, and *ALPL* in human osteoblasts in response to different culture conditions (n = 4). Statistical significance (*p* ≤ 0.05) is represented as relative to control (*) and between treatments (#).

The QC assessment of purified extracts revealed that all the samples showed sufficient RNA quality (260/280 ratio = 1.9–2.1; 260/230 ratio = 2.1–2.2) and quantity (10–30 ng/µL) for RT-qPCR applications. As recommended by the MIQE guidelines [68], efficiency values were also acceptable for all the primers assayed (ERunx2 = 87.3%; r2 = 1.0, ESP7 = 94.3%; EALPL = 97.0%; r2 = 1.0, EEif2B1 = 87.4%; r2 = 1.0; EYWHAZ = 87.4%; r2 = 1.0). A box plot summarizing the distribution of the log relative gene expression (logRGE) of *Runx2*, *SP7*, and *ALPL* is represented in Figure 4. The Livak method or 2^−ΔΔCt^ method (including slightly improved versions) is the most commonly used way for qPCR data analysis [66,69,70]. However, before applying the classic Livak method, certain criteria should be fulfilled. The first assumption is that both target and reference genes should be amplified with efficiency near 100% and within 5% of each other. As shown by the range of calculated PCR primer efficiency, this assumption was not met in the present study. Moreover, it should be noted that this method was developed to compare the expression of a target gene by using a single reference gene. Unfortunately, the Pfaffl model [67], which constituted an improvement over the classical 2^−ΔΔCt^ method, also cannot deal with multiple reference genes, which is required for reliable measurements of subtle expression differences. Depending on the method used, normalized relative quantities (NRQs) are usually expressed as relative gene expression ratio (R in the Pfaffl method), and 2^−ΔΔCt^ or log(2^−ΔΔCt^) in the Livak method. By contrast, in the present study, two different reference genes were included in order to increase resolution and accuracy, since the use of only one may lead to relatively large errors [71,72]. During recent decades, different methods that allow for the inclusion of multiple reference genes have been developed, quenching occasional regulations of single reference genes and greatly stabilising gene expression levels. For example, Riedel et al. published a modified version of the 2^−ΔΔCt^ method dealing with multiple reference genes [73]. In this sense, the method selected during this experiment for gene expression analyses by the geometric averaging of multiple internal control genes was developed by Vandesompele et al. [58] and implemented later by Hellemans et al. [59] in the qBase framework. This method is based on the Pfaffl model, but the relative gene expression is calculated by using the geometric averaging of all the relative quantities of the multiple reference genes included in the experiment, and results regarding NRQs are computed as relative gene expression levels (according to the notation used in this experiment, relative gene expression or RGE) as reported by Vandesompele et al. [58], Hellemans et al. [74], and Poppe et al. [75].

In accordance with the Kruskal–Wallis test, significant differences in logRGE of *Runx2*, *SP7*, and *ALPL* were detected at *p* ≤ 0.05. Compared with the control group, the logRGE of *Runx2* increased significantly (4-fold higher expression than in the calibrator) in osteoblasts cultured under both experimental conditions, i.e., PRGF supernatant (*p* = 0.018) and PRGF clot (*p* = 0.018), while that of *SP7* increased significantly in osteoblasts grown in PRGF clot (8.6-fold higher expression than in the calibrator; *p* = 0.036). On the other hand, the addition of PRGF supernatant to osteoblast growth medium induced *ALPL* up-regulation (7.0-times more expression than in the calibrator; *p* = 0.022). In this sense, the replacement of FBS with the PRGF supernatant in the culture medium promoted osteoblast differentiation, bone morphogenesis, and bone matrix mineralization in primary human osteoblast cultures, as revealed by the *Runx2* and *ALPL* relative expression profiles. Similar effects were observed in *Runx2* transcription regulation when the PRGF clot was included. Moreover, the *SP7* transcription factor, which plays an important role in both driving the differentiation of mesenchymal precursor cells into osteoblasts or osteocytes and also in inhibiting chondrocyte differentiation, showed elevated relative expression levels in PRGF clots. 

#### 3.4.2. ALPL Activity Determination

**Figure 5:**
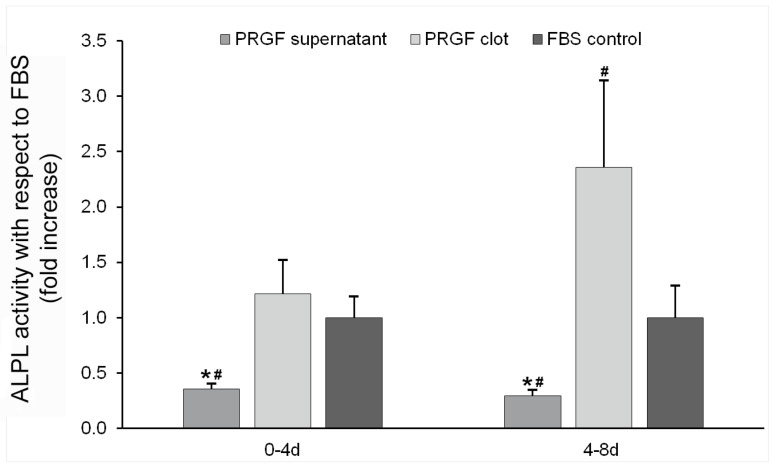
ALPL activity determination in the culture medium conditioned by bone cells. ALPL enzymatic activity in the extracellular milieu of bone cells treated with PRGF formulations (n = 4). Results expressed as fold increases with respect to the data obtained for the control condition (osteoblasts cultured on bare plastic surface with ObM supplemented with 15% FBS (*v*/*v*)). * Statistically significant differences with respect to the PRGF clot (*p* ≤ 0.05). ^#^ Statistically significant differences with respect to the control condition for the same study period (*p* ≤ 0.05).

ALPL enzymatic activity was determined in the culture medium conditioned by bone cells treated with both supernatant and clot PRGF formulations. The results obtained in milliunits per well were finally expressed as fold increases in activity with respect to the control condition (cells seeded on bare plastic surface and treated with ObGM). Significantly higher enzyme activity was detected for the PRGF clot in comparison with the supernatant formulation in two of the periods analysed (1.21x ± 0.30 vs. 0.36x ± 0.04, and 2.36x ± 0.79 vs. 0.29x ± 0.05, for clot and supernatant, respectively, at 4 and 8 days) (Figure 5).

## 4. Discussion

Osteoblast cells are derived from pluripotent bone marrow stem cells (BM-MSCs). During in vitro differentiation from osteoprogenitors into mature osteoblast cells, three phases can be distinguished in relation to the different stages of cell maturation. Firstly, a proliferative phase is associated to the more undifferentiated cell phenotype. As a higher degree of differentiation is acquired, the constituents of the bone matrix are synthesized. Finally, the ECM becomes mineralized by osteoblasts in late differentiation stages. The expression of osteoblast markers such as ALPL and type I collagen starts earlier in the immature osteoblasts, and it is maintained throughout the whole differentiation process in addition to other bone proteins associated with late stages of maturation, such as OPN, OC, and bone sialoprotein II (BSP-II). However, the coexistence of 3 proteomic profiles revelling their potential differential roles has recently been described by Gong et al. in freshly isolated osteoblast populations without any in vitro culturing [76]. Thus, undetermined osteoblasts, preosteoblasts, and mature osteoblasts were characterized. Despite the fact that quantitative cell characterisation has not been performed, ALPL activity, and OC and OP markers were detected in almost all the cells of the bone population isolated and maintained in ObGM culture medium and on plastic surface, which would suggest it mainly consisted of mature osteoblasts.

Regarding proliferation, it was found to be significantly higher for the PRGF supernatant compared with the clot formulation and the control condition. PRGF releasate consists of a pool of growth factors, cytokines, and biologically active molecules whose proliferative effect on bone cells has been widely confirmed [40,77,78]. Higher proliferation rates in association with early phases of the osteogenic process have already been described [79].

*Runx2* is one of the most well-studied transcription factors that control osteoblast differentiation. While weak *Runx2* expression is associated with uncommitted bone cells, its maximum expression level is reached in the early stages of osteogenic differentiation, being later down-regulated in mature osteoblasts [8,80,81,82]. Our data show that *Runx2* expression was drastically increased by the treatment of the isolated bone cells with both PRGF preparations. These results could suggest a reversion of the overall stage of differentiation by promoting the immature osteoblast subpopulation in osteoblast cells located both in contact with the PRGF clot and at a distance from it. Furthermore, the effect of platelet-rich derivatives on *Runx2* expression in bone cells, including alveolar bone cells [83] and osteoblasts [77,84,85,86], has been highlighted by many authors. Similar findings regarding the *Runx2* expression promoting effect of platelet-rich fibrin (PRF) in mesenchymal stem cells have been published recently [87,88]. Moreover, platelet-rich fibrin also increases BMP2 expression via the activation of the transforming growth factor beta (TGF-β) signalling pathway in oral fibroblasts [89]. Despite the fact that the specific mechanisms underlying the regulation of osteoblast differentiation by platelet-rich derivatives are still incompletely understood, TGF-β1 and BMP2 may play a key role in *Runx2* up-regulation. According to Lee et al., *Runx2* is a common target gene of TGF-β and BMP signalling pathways [90,91]. In this sense, either TGF-β1 or BMP2 can lead to *Runx2* up-regulation, which induces the expression of matrix gene products and suppresses myoblast determination protein 1 (MyoD) expression in pluripotent mesenchymal precursor cells, but not osteoblast-specific gene expression patterns [90]. Moreover, Kawane et al. [92] concluded that *Runx2* enhances the FGF2-induced proliferation of osteoblast progenitors and also the proliferation mediated by the fibroblast growth factor receptors Fgfr2 and Fgfr3. All these results could newly suggest a lower level of differentiation in bone cells at distant locations induced by the clot releasate. 

*SP7* is a zinc finger-containing transcriptional factor which runs downstream of *Runx2* to promote osteoblast maturation [93]. Yoshida et al. summarized the stage-dependent regulation of *SP7* expression and protein activity during osteoblast differentiation [94]. In fact, *SP7* inactivation in mice disrupts osteocyte maturation and functionality, and also leads to a lack of bone formation and irregular accumulation of unresorbed calcified cartilage beneath the growth plate [95]. *SP7* mRNA expression is induced directly via Msh homeobox 2 (Msx2) and indirectly through BMP2 mediated by *Runx2* and distal-less homeoboxes DLX3 or DLX5 transcription factors [96,97]. In our research, when *SP7* gene expression was analysed, a statistically significant higher level of expression was detected for cells cultured onto the PRGF clot. In this sense, Goto et al. found significantly higher levels of *SP7* mRNA in PRP/osteoblast complexes than in PPP/osteoblast complexes after 7 days [98]. Mokthari et al. also reported increased *osterix* mRNA levels in PRP-treated osteoblasts in the presence of a three-dimensional scaffold [99], while Li et al. found that PRF exudates promote *osterix* expression in human periodontal ligament cells [100]. Although, in our study, *Runx2* showed similar expression profiles in both treatment groups, up-regulated *SP7* was detected only in the PRGF clot group. As a consequence, it could be hypothesised that the different PRGF effects on *SP7* expression patterns depending on the osteoblast proximity to the PRGF clot could involve other transcription factors, including Msx2, DLX-3, or DLX-5. Nevertheless, the scientific bibliography regarding the effect of platelet-rich derivatives on the expression of these genes is scarce. For example, Hamdan et al. reported that Msx2 expression in rat osteoblastic cells cultured with 10% of platelet-poor plasma (PPP) was significantly higher than in those cultured with 10% FBS [101]. The initial expression of the *Runx2* transcription factor in immature bone cells and, afterwards, the expression of both master osteoblast differentiation regulators, *Runx2* and *SP7*, have been already described [21,23,102]. The latter has been confirmed to be involved in osteoblast differentiation, maturation, and cell activity [93,103]. Thus, it might be assessed that the PRGF clot would support a more advanced differentiation state when the bone cell population is in contact with it than in the case of being at a distant location. In this regard, a remodelling phenotype of osteoblast cells in which active synthesis of type I collagen was promoted by culturing them onto a PRGF clot has been already described in previous research [104]. Moreover, several in vivo studies have confirmed the promotion of the osteogenic regenerative potential of mesenchymal stem cells (MSCs) by combining them with PRP carriers [105,106,107,108,109,110].

Concerning the ALPL enzyme, its expression in bone cells treated with PRGF supernatant was significantly increased compared with the PRGF clot and the control condition. According to the consulted bibliography, different platelet-rich derivates, either alone or combined, enhance the expression of *ALPL* and induce mineralization in osteoblasts and other oral cell types [85,98,111,112,113], but the observed effect on ALP activity is formulation-dependent [114,115]. This pattern of expression is not the expected, considering those opposite results obtained for the *SP7* transcription factor. Therefore, the statistically significant higher proliferation rate and the lower *SP7* expression level obtained for the treatment with the PRGF supernatant compared with the PRGF clot formulation could lead to suspecting decreased values for the MEC mineralization-related enzyme. As shown by the present data, both formulations showed a tendency towards an increase in *Runx2* expression, a transcription factor that controls osteoblast differentiation and that reaches its maximum expression level in the early stages of differentiation. On the other hand, the *SP7* transcription factor, which runs downstream of *Runx2* to promote osteoblast maturation, was significantly up-regulated in osteoblasts grown on PRGF clots. As a consequence, it could be hypothesised that the differential effect of PRGF derived-formulations observed on *SP7* expression patterns could involve other transcription factors, including Msx2, DLX-3, or DLX-5. We hypothesise that the sustained *SP7* overexpression in osteoblasts grown onto PRGF clots inhibited osteogenic differentiation in a late stage [94]. Thus, *ALPL* mRNA expression, which could be considered an osteoblastogenic marker associated with mature osteoblasts [82], was significantly increased in PRGF-supplemented osteoblasts, but not in osteoblasts grown on PRGF clots. On the other hand, surprisingly, ALPL activity was found to be diminished in osteoblast cells treated with PRGF supernatant but was significatively much higher in bone cells cultured on PRGF clots. These apparently contradicting results could be explained by the fact that *ALPL* encodes a preprotein that should be proteolytically cleaved, resulting in a signal peptide and a proprotein that are subsequently processed to generate the active mature enzyme [116]. The possibility of any alteration in the preprotein activation process, like the inhibition of the enzymes involved in ALPL maturation, could be a possible explanation [117]. However, the correspondence between mRNA expression and ALPL activity has been widely confirmed [118,119]. ALPL is a membrane-bound glycoprotein, but its hydrolytic activity can be also detected in two cell surface-independent forms: an anchorless soluble protein generated by the proteolytic action of specific hydrolases and an anchor-intact insoluble form produced by membrane fragmentation and/or the exocytosis of membrane vesicles [120,121,122,123,124,125,126]. Moreover, ALPL activity associated with osteoblast-derived exosomes has been recently described [127]. In this sense, the ALPL activity in the culture medium conditioned by osteoblast cells was determined, finding, in this case, the expected values according to *SP7* gene expression. Thus, ALPL activity quantification showed significant lower levels for the treatment with the PRGF supernatant in the longest period analysed compared with the clot formulation and the control condition. 

Despite the fact that the experimental limitations should be considered, such as the number of donors of biological samples, in conclusion, PRGF could modulate the differentiation status of osteoblasts in a different way depending on their proximity to the fibrin clot. Thus, the PRGF clot releasate could support a higher proliferation rate in osteoblasts at a distant location, in addition to reduced *SP7* gene expression and diminished ALPL activity, which would promote a more undifferentiated stage of this bone cell population. Contrarily, PRGF clots could contribute to a more advanced stage in the osteogenic process of the osteoblasts located in intimate contact with the clot surface. Future research could consider further analysis of the specific factors contained in the PRGF clot that promote a more or less differentiated subpopulation of osteoblastic cells.

## Figures and Tables

**Table 1:** Haematological characterisation. Platelet, leukocyte, and erythrocyte counts in peripheral blood and PRGF used for supernatant and fibrin clot preparation. * Folds with respect to peripheral blood.

Sample	Platelet Count (×10^6^/mL)	Leukocyte Count (×10^6^/mL)	Erythrocyte Count (×10^9^/mL)
Peripheral blood	176	6.1	4.59
PRGF	391 (2.2 x) *	0.2 (0.03 x) *	0.01 (0.00 x) *

## Data Availability

The raw data supporting the conclusions of this article will be made available by the authors on request.
